# Does visualisation during urethrocystoscopy provide pain relief? Results of an observational study

**DOI:** 10.1186/s12894-015-0053-2

**Published:** 2015-07-01

**Authors:** J. Koenig, S. Sevinc, C. Frohme, H. Heers, R. Hofmann, A. Hegele

**Affiliations:** Department of Urology and Pediatric Urology, University hospital Marburg, Philipps University, Marburg, Germany

**Keywords:** Cystoscopy, Pain, Bladder, Real-time visualisation

## Abstract

**Background:**

To measure the effects of real-time visualisation during urethrocystoscopy on pain in patients who underwent ambulatory urethrocystoscopy.

**Methods:**

An observational study was designed. From June 2012 to June 2013 patients who had ambulatory urethrocystoscopy participated in the study. In order to measure pain perception we used a numeric rating scale (NRS) 0 to 10. Additional data was collected including gender, reason for intervention, use of a rigid or a flexible instrument and whether the patient had had urethrocystoscopy before.

**Results:**

185 patients were evaluated. 125 patients preferred to watch their urethrocystoscopy on a real-time video screen, 60 patients did not. There was no statistically relevant difference in pain perception between those patients who watched their urethrocystoscopy on a real-time video screen and those who did not (p = 0.063). However, men who were allowed to watch their flexible urethrocystoscopy experienced significantly less pain, than those who did not (p = 0.007). No such effects could be measured for rigid urethrocystoscopy (p = 0.317). Furthermore, women experienced significantly higher levels of pain during the urethrocystoscopy than men (p = 0.032).

**Conclusions:**

Visualisation during urethrocystoscopy procedures in general does not significantly decrease pain in patients. Nevertheless, men who undergo flexible urethrocystoscopy should be offered to watch their procedure in real-time on a video screen. To make urethrocystoscopy less painful for both genders, especially for women, should be subject to further research.

## Background

Pain relief is one of the most important tasks of every doctor. It is one of the most common reasons for consultation and a basic skill of the medical profession. Therefore, pain relief is something patients expect from their doctors. Unfortunately, it is also one of the most complicated tasks and often the best medical practice requires procedures that are not only uncomfortable, but inflict pain.

One such a procedure is the urethrocystoscopy. It is not invasive enough to justify the risk of complete anaesthesia but also too uncomfortable to be not taken seriously. As a frequently used tool in diagnosis and a treatment of various urological conditions such as bladder carcinoma, tumour infiltration and haematuria, the significance of urethrocystoscopies in everyday urological practice has increased [1].

Various attempts have been made in the past to make urethrocystoscopy less painful. The biggest achievement in patient comfort probably presents the invention of the flexible urethrocystoscope. Before, men and women alike were examined with a rigid instrument. Due to gender related anatomical differences rigid urethrocystoscopy on men was quite painful [2]. This changed with the invention of the flexible instrument. Since then repeated effort was made to further improve patients comfort. The application of lidocaine gel was introduced. Unfortunately, its benefit remains uncertain [3]. Several other attempts to reduce pain during uerthrocystoscopy have been made, as a technique called a bag squeeze [4], the inhalation of nitrous oxide gas during the procedure [5], application of Midazolam [6] or transcutaneous electrical nerve stimulation [7].

Over the past few years evidence occurred that patients might benefit from watching their urethrocystoscopy real-time on a video screen [8–11].

Up to now only specific parts of the patient population were investigated. Men who were to undergo flexible urethrocystoscopy and women who had rigid urethrocystoscopy. Results of previous studies show [8–11], that visualisation during urethrocystoscopy decreases pain in certain patient populations in randomised controlled trails. As pain is a composite of an entire experience influenced by many different factors, we wanted to test the outcomes in every day clinical practice.

We generated a prospective observational study in everyday clinical practice to measure the pain patients experienced during urethrocystoscopy while visualising the procedure on a real-time video screen compared with the pain patients who did not visualise their procedure experienced. The aim was to test whether visualisation aided in increasing patient comfort during urethrocystoscopy.

We expected that distraction would decrease the level of pain patients experienced during the procedure [12].

## Methods

### Observational study design

To access the differences in pain perception between patients who were able to watch their urethrocystoscopy real-time on a video screen and patients who were not, we conducted a prospective observational study. Our aim was to stay as close as possible to everyday clinical practice to provide a clear image of patients’ pain perception in an everyday clinical setting. Everyday clinical practice is influenced by many different factors which have to be eliminated in a randomised controlled trial. Therefore what has been evaluated in a randomised controlled study cannot necessarily be applied to everyday clinical practice. Hence, we decided not to randomise our study. As the study was done to investigate clinical data of patients treated solely at our institution, and data obtained was anonymized concurrently, approval of the local ethics committee (Ethics Committee, Faculty of Medicine Marburg) was therefore not required according to the German Ethics Committees regulations.

### Patients

The patient population consisted of all patients who underwent an ambulatory urethrocystoscopy from June 2012 to June 2013 in the urological outpatient clinic of the Department of Urology and Paediatric Urology at the university hospital of Marburg. Exclusion criteria were the necessity of systemic or local anaesthesia and the use of sedatives or analgesics other than Instillagel®. In total 185 patients were included.

### Urethrocystoscopy

The procedure patients received was not randomized. They could choose whether they wanted to visualise the procedure on a real-time video monitor along with the urologist or whether they preferred not to.

Pain perception and pain control depend on various factors and differ greatly between individuals [12]. To take individual differences in account we decided to let the patient choose which option he or she felt more comfortable with. The monitor was then positioned according to patient’s wishes.

The urethrocystoscopy was performed according to the standardised scope technique of the Department of Urology and Paediatric Urology of the University hospital, Marburg. The procedure was performed in lithothomy position. Pelvis and legs were covered and anus and genitals were disinfected. For rigid urethrocystoscopy Storz, Wolf or Olympus 70/120° instruments were used and the flexible urethrocystoscopy was also performed with Storz, Wolf and Olympus instruments.

Instillagel® was applied 10 min before the procedure as recommended in recent studies [3]. In male patients a penile clamp was used to ensure proper instillation.

In order to avoid limitations in our results based on the skill of a single urologist, the urethrocystoscopy was performed by different urologists. Therefore interindividual differences in technique and skill were taken into account, as they are a part of everyday clinical practice.

### Data

After the procedure patients were asked by the attending nurse to quantify their pain on a numeric rating scale (NRS) from 0 to 10. 0 was defined as no pain, 1–4 mild pain, 5–7 moderate pain and 8–10 as severe pain [13].

Additional data was collected including the patients gender, reasons for intervention, the use of a rigid or flexible instrument, whether the patient had had urethrocystoscopies before and what kind of instrument was used. Furthermore we assessed whether patients thought themselves to be sensitive to pain or no. All data was computerised on Microsoft Excel® 2013 (Microsoft, USA) and analysed with IBM SPSS® Statistics for Windows Version 22 (Ehningen, Germany) using the Mann – Whitney – U – test. A *p* value of < 0.05 was considered statistically significant.

## Results

### General Data

185 patients were evaluated (age 17 to 91 years). The mean age of the 44 female patients was 59 years (17 – 91 years). The mean age of the 141 male patients was 65 years (26 – 89 years). 125 patients (66 %) wanted to follow their urethrocystoscopy in real – time on video with the urologist, 60 (34 %) patients preferred not to. 131 had flexible urethrocystoscopies. 91 (69,5 %) of these patients watched their procedure real-time and 40 (30,5 %) did not. Of the 54 patients who underwent rigid urethrocystoscopy 34 (63 %) wanted to watch the procedure on the video screen and 20 (37 %) did not. 105 (55 %) patients had undergone an urethrocystoscopy before and for 80 (45 %) patients it was the first time they had had the procedure. Patient data is summarised in Table 1.

**Table 1:** Summarized patient data

	Number	Average age(range)	Flexible/rigid	Video yes/no	Experience yes/no	Tumor/follow up	Prone to feel pain yes/no
Total	185	64(17–91)	127/54	125/60	103/82	97/88	43/142
Men	141	69 (26–89)	125/14	100/42	85/56	82/59	29/112
Women	44	64 (17–91)	2/41	25/17	18/26	15/29	14/30

### Pain

The median value on the numeric rating scale was 2 (±2.2, range 0–9) for patients who watched their procedure in real-time on a video screen and 3 (±2.1, range 0–9) for those who did not without statistical difference (p = 0.063, see Table 2).

**Table 2:** Influence of visualisation/no visualisation on individual pain scores separated by kind of urethrocystoscopy

	Visualisation (n)	No visualisation (*n*)	*p* value
Urethrocystoscopy in general	125	60	0.063
	NRS = 2	NRS = 3	
Flexible urethrocystoscopy	91	40	0.007
	NRS = 2	NRS = 3.5	
Rigid urethrocystoscopy	34	20	0.317
	NRS = 3	NRS = 3	

**Fig. 1:**
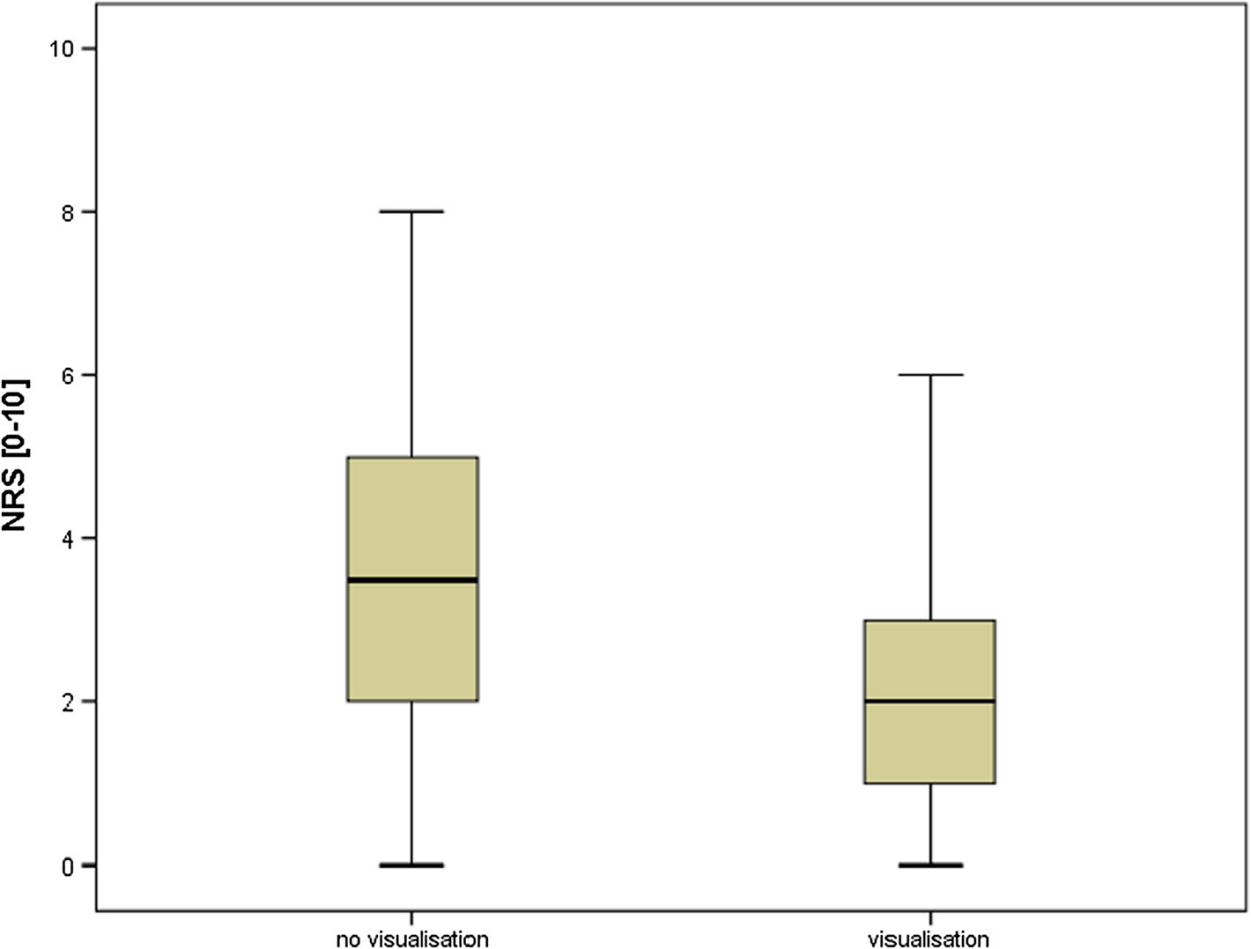
Significant different pain scores in patients undergoing flexible urethrocystoscopy with or without visualisation (p=0.007)

When analysed separately patients who had flexible urethrocystoscopies, had significantly less pain when they were allowed to watch the procedure (2 (±2.0, range 0–8) vs 3.5 (±2.1, range 0–8), p = 0.007; see Fig. 1).

When using the rigid urethrocystoscope no significant differences could be shown.

Experience with the procedure proved to be significant in pain perception. Patients who had had the procedure before had significantly less pain (p < 0.005). Women who had had experience with urethrocystoscopy still had significantly more pain than their male counterparts (p = 0.031). Pain perception was not significantly lower in patients with experience who watched the procedure than in those who did not (p = 0.165).

### Gender differences

Of the 127 patient who had flexible urethrocystoscopies only 2 (1.6 %) were women. Therefore, no valid results could be obtained about the benefit of real-time visualisation in women who had flexible urethrocystoscopies. In contrast, 41 patients who had rigid urethrocystoscopies were women (74,5 %) and 14 were men.

Analysed separately women who could watch their rigid urethrocystoscopy on a real-time video screen did not feel significantly less pain than women who did not (3 vs 2, p = 0.290). No valid results could be obtained regarding men who had rigid urethrocystoscopies, as only two men decided not to watch their urethrocystoscopy.

**Fig. 2:**
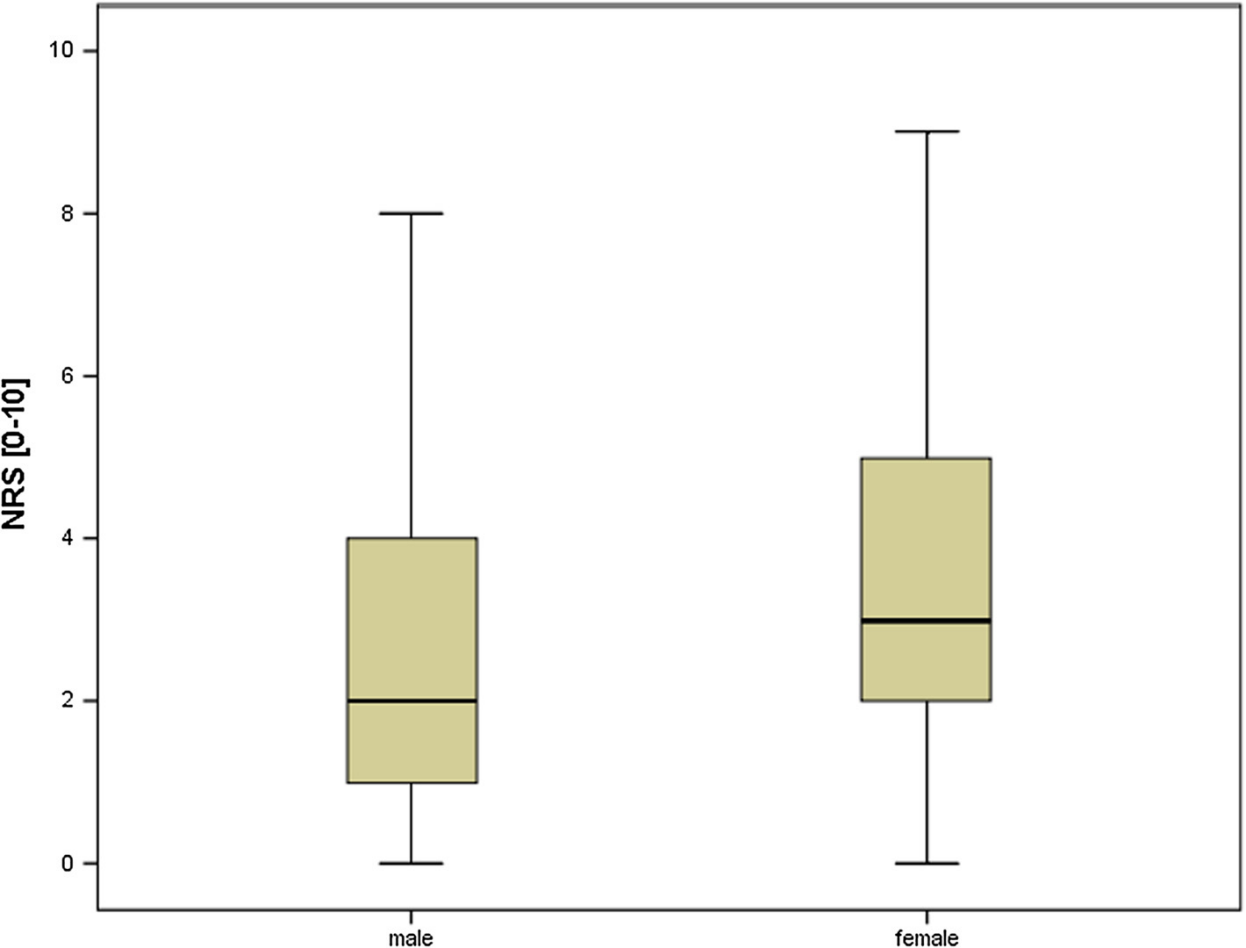
Female patients had signficant more pain during urethrocystoscopy than male patients (p=0.032)

Female patients (3.5, ± 2.4, range 0–9) had significantly more pain than male patients (2.7, ± 2.2, range 0–9) during urethrocystoscopies (p = 0.032, see Fig. 2), although there was no significant difference in the self – assessment of pain between both genders (p = 0.092).

## Discussion

In our observational study we investigated the differences in pain during urethrocystoscopy between patients who could watch their procedure on a real-time video screen and patients who could not in an everyday clinical setting. We found that only men who underwent flexible urethrocystoscopy and wanted to watch their procedure on a real-time video screen perceived their urethrocystoscopy as significantly less painful. For other patients this effect was not observed. Furthermore, women who underwent urethrocystoscopy have significantly more pain than men.

To our knowledge only a few studies so far have examined the impact of real – time visualisation on pain perception during urethrocystoscopy. Clement et al. [8] conducted a prospective randomized study in 2004 with patients who had flexible urethrocystoscopy. Four further randomised studies on male patients who underwent flexible urethrocystoscopy were conducted by Patel [9] in 2006, Cornel [14] in 2007, Soomro [10] in 2010 and Zhang [11] in 2011. Patel also analysed the impact of real – time visualisation on pain in women who underwent rigid urethrocystoscopies in another randomised study in 2008 [15].

So far only Clements et al. [8] showed the impact of real – time visualisation on urethrocystoscopy on patients of both genders.

A number of differences between the randomised controlled studies and this observational study make direct comparison difficult.

First, in our study we used a numeric rating scale 0 to 10 to measure perceived pain. Patel et al. used a 100 mm visual analogue scale in both of his studies [9, 15]. Clements et al., Cornel et al., Soomro et al. and Zhang et al. used a visual analogue scale 0 to 10 [8, 10, 11, 14]. According to Williamson et al.’s review on pain scales both scales provide the same information but cannot be converted into one another [16]. Given that the different scales transfer the same information the results of the different studies can be compared.

Second, inclusion and exclusion criteria were different in the studies mentioned. In order to best approximate everyday clinical practice our exclusion criteria were broadly defined as the use of general or local anaesthesia and analgesics. In the other studies exclusion criteria were more narrowly defined [8–11, 14, 15].

Third, different numbers of urologists performed urethrocystoscopies on the included patients. In Soomro study and our study more than one urologist performed the urethrocystoscopy, so that the results would not be limited to the skill of one person [10]. In Clements et al., Patel et al. and Zhang et al. studies only one urologist performed the procedure [8, 9, 11]. We think both approaches could include possible bias. Assessing the results of more than one urologist mixes different skills and difference in performance which might influence pain. Limiting the results to the performance of one urologist means that the pain score can only be related to this one urologist’s performance. We decided to have more than one urologist perform urethrocystoscopy as it is closer to everyday clinical practise, even though we are aware that this approach might include possible bias. As this study was done in an outpatient clinic to assess pain during urethrocystoscopy in everyday clinical practise, we believed the results could be better related to everyday clinical practise if more than one urologist performed the procedure.

The greatest limitation of our study is that it was not randomised. Patients could choose whether they wanted to observe their own urethrocsytoscopy or not. Pain is a very subjective parameter. Therefore, it is difficult to assess and to generalise [17]. We tried to create the most comfortable setting for our patients, which included letting them choose what they felt most at ease with. In letting the patient choose whether they wanted to watch the procedure on a real-time screen, we were closer to everyday clinical practice, where no patient who does not want to watch his or her own procedure on real time video is forced to do so.

Our data showed that visualisation on a real-time screen does not make urethrocsytoscopy less painful for patients in general. Kesari et al. investigated the role of visualisation on a real-time video screen with thorough explanation during the procedure [18]. In this small cohort (n = 51) patients who watched their procedure real time on a video screen did not benefit regarding their anxiety or their pain. In this study visualisation was examined as an addition to explanation and not as a measure to decrease pain on its own [18].

Focusing on flexible urethrocystoscopies our results confirm the results found by Clements et al., Patel et al., Soomro et al. and Zhang et al. [8–11]. Men who are able to watch their urethrocystoscopy in real-time on a video screen find it significantly less painful. Cornel et al.’s results oppose these findings [14]. Soomro et al. suggested that this might be due to the fact that the lithotomy position is less comfortable than the supine position [10] In Zhang et al.’s study and in our observational study all patients were examined in the lithotomy position, nevertheless our results showed that the procedure was significantly less painful for those who could watch it on a real – time video screen. Cultural differences between European and American men are offered as a possible explanation [11]

In our patient cohort only 2 women had flexible urethrocystoscopy. Therefore based on this data no assumptions can be made about the effect real time visualisation has on pain during flexible urethrocystoscopy on women. This might be due to the recommendations regarding flexible urethrocystoscopy on women based on the recent findings of Gee et al. and Quiroz et al. [19, 20]. They state that there is no difference in comfort for women regardless of whether or not a rigid or a flexible instrument is used. For rigid urethrocystoscopies our results concerning women were similar to those of Patel et al. regarding the use of real-time visualisation in women who had rigid urethrocystoscopies. It has to be pointed out, that our observational study also included men who had rigid urethrocystoscopies. But as only two men did not watch their rigid urethrocystoscopy on a real-time video screen, no valid statistical analysis could be performed.

Cornel et al. found that patients who had experience with urethrocystoscopy did not find it more comfortable than those who had not [14]. We found that patients who had had urethrocystoscopy before had significantly less pain than those who did not. These finding are in line with recently published data in over 1300 consecutive procedures showing that pain levels during first cystoscopy is higher than for repeated cystoscopies [21]. Again, this might be due to cultural differences but it could also be due to the smaller number of patients Cornel et al. included in their study. Anatomy suggests that urethrocystoscopy should be more painful for men than for women given the difference in length of the urethra. In our observational study this was not the case. Women experienced significantly more pain than men. This suggests that the invention of flexible urethrocystoscopy made the procedure more comfortable for men. No such benefit occurred in women. Patel et al. presume that this is the case because women are not able to visualise the most painful part of urethrocystoscopy: the passage of the urethra as it is performed blindly in women [15]. It is however not clear what the most painful part in urethrocystoscopy on women is, because the study generated by Taghizadeh et al. included only men [22]. To our knowledge, no such study about the most painful part in urethrocystoscopy in women exists. As Patel et al. points out, there is probably more than one factor that makes urethrocystoscopy more painful for women [15]. The results of a recent study suggest that by using flexible instruments it is possible to reduce pain in women during flexible urethrocystoscopy [21]. Therefore the use of a flexible instrument on women should be encouraged.

## Conclusions

In regard to pain prevention in urethrocystoscopy three valuable conclusions can be drawn from this study. First, men who undergo flexible urethrocystoscopies should be offered to watch their procedure in real – time on video along with the urologist. If they agree to watch they will benefit from their decision. Second, more effort should be put into making urethrocystoscopy more comfortable for women, especially because female patient’s pain threshold might be lower than the pain threshold of male patients [17]. It is therefore important that sufficient pain control is administered on both genders. Third, real-time visualisation alone does not make the urethrocystoscopy less painful. On its own it is not sufficient enough.

New efforts should be made to improve patients’ comfort level during urethrocystoscopy procedure. An example of such efforts can be found in the new observations made by Yeo et al. and Zhang et al. who found that using music to help patients relax during urethrocystoscopy may lead to benefical results [23, 24].
